# Re-irradiation of recurrent pediatric ependymoma: modalities and outcomes: a twenty-year survey

**DOI:** 10.1186/s40064-016-2562-1

**Published:** 2016-06-24

**Authors:** Maria Jesus Lobón, Francisco Bautista, François Riet, Frederic Dhermain, Sandra Canale, Christelle Dufour, Thomas Blauwblomme, Michel Zerah, Kevin Beccaria, Christian Saint-Rose, Stephanie Puget, Christian Carrie, Eric Lartigau, Pierre-Yves Bondiau, Dominique Valteau-Couanet, Jacques Grill, Stephanie Bolle

**Affiliations:** Department of Pediatric and Adolescent Oncology, University Paris Sud Villejuif, Gustave Roussy, 114 Rue Edouard Vaillant, 94805 Villejuif, France; Department of Radiotherapy, University Paris Sud Villejuif, Villejuif, France; Department of Radiology Gustave Roussy, University Paris Sud Villejuif, Villejuif, France; Department of Neurosurgery, Hôpital Necker Enfants-Malades, Paris, France; Department of Radiotherapy, Centre Léon Bérard, Lyon, France; Centre Oscar Lambret, Lille, France; Centre Antoine-Lacassagne, Nice, France

**Keywords:** Ependymoma, Recurrent, Radiotherapy, Radiosurgery, Children

## Abstract

**Background:**

Standard treatment for recurrent ependymomas is not defined. Re- irradiation has been proposed but its modalities and results are still to be explored.

**Patients and methods:**

From June 1994 to December 2013, 32 pediatric patients with ependymoma were re-irradiated for local (n = 15) or metastatic (n = 17) relapses. Files were reviewed retrospectively.

**Results:**

Local relapses were treated with hypofractionated focal radiotherapy (hypoFFRT) (n = 8) or focal fractionated radiotherapy (FFRT) (n = 7). Metastatic relapses were treated with hypoFFRT (n = 3), FFRT (n = 3), spinal radiotherapy (n = 4) and craniospinal irradiation (CSI) (n = 7). Median PFS and OS after re-irradiation were 1.2 and 3.5 years respectively with a median follow-up of 2.1 years (0.2–11.4). For local relapses, median PFS was 2.5 years for patients treated with hypoFFRT versus 1.2 years for patients treated with FFRT (p = 0.2). For metastatic relapses, median PFS was 0.7 years for patients treated with focal radiotherapy (hypoFFRT, FFRT, spinal radiotherapy) versus 6.8 years for patients treated with CSI (p = 0.073). 15 patients achieved greater PFS after second radiotherapy (RT2) than after first radiotherapy (RT1). 27 patients (84 %) had surgery before re-irradiation. PFS was better for patients with GTR before RT2 (14.7 vs 6.7 months) (p = 0.05). 5 patients developed radionecrosis; only one required corticosteroids.

**Conclusion:**

Re-irradiation at relapse is a safe, feasible and potentially curative treatment. Metastatic relapse may require CSI even when isolated and re-operated. For local relapses, considering conflicting results in the literature, a randomized trial is warranted to explore fractionated focal radiotherapy versus hypofractionated focal irradiation.

## Background

Ependymomas are the third most common type of brain tumor in children. About 90 % are found in the intracranial region, 80 % in the posterior fossa (Merchant et al. 2009). More than 50 % are younger than 5 years. Overall prognosis is dismal with a 5 year overall survival and progression free survival rate of 56–85 and 38–74 % respectively (Merchant et al. 2009; Rousseau et al. 1994; Pollack et al. 1995).

Upfront adjuvant radiotherapy has become the mainstay of the treatment in children above the age of 36 months because chemo resistance to most of the known agents is the rule (Merchant et al. 2009; Massimino et al. 2011; Garvin et al. 2012) .

Treatment strategies used to be stratified on age and on surgical results but radiotherapy is now considered as the first line of treatment in younger children. At diagnosis standard treatment for older patients with ependymoma includes maximal surgical resection and adjuvant focal radiotherapy. For younger patients, deferring or omitting radiotherapy has been attempted in various protocols (Merchant et al. 2009; Garvin et al. 2012; Grill et al. 2001; Grundy et al. 2007; Duffner et al. 1993, 1998) but the efficacy of radiotherapy-first approaches even in young children has lead the investigators to lower the age limit to start radiotherapy (Merchant et al. 2009). Despite these attitudes, a significant number of children with ependymoma relapse (Messahel et al. 2009).

Recurrence after focal radiotherapy is local in 39 % of the patients, metastatic in 41 % and combined in 19 % (Merchant et al. 2009). The unequivocally recognized risk factor for relapse is the absence of complete surgery.

Management of recurrent ependymoma is not standardized. Compared with chemotherapy, survival seems to be dramatically improved by re-irradiation (Bouffet et al. 2009, 2012). In some reports, re-irradiation could be curative for recurrent ependymoma in cases where complete resection can be offered (Merchant et al. 2008). Different radiotherapy modalities are used: focal fractionated radiotherapy (FFRT) (that includes 3D conformal radiation therapy, intensity modulated radiation therapy and proton therapy), hypofractionated irradiation (hypoFFRT) (that includes stereotactic radiosurgery and hypofractionated stereotactic radiotherapy) and craniospinal irradiation (CSI) (Merchant et al. 2008; Hoffman et al. 2014; Stauder et al. 2012; Kano et al. 2010; Krieger and McComb 2009). Toxicity of re-irradiation in selected indications is manageable (Bouffet et al. 2012; Merchant et al. 2008; Lo et al. 2006). However, no strategy has been clearly established for these patients.

We aim to better understand the evolution of re-irradiated patients to define at recurrence the best indications of the different radiotherapy modalities.

## Patients and methods

### Patients

Between June 1994 and December 2013, all children with a relapsed ependymoma re-irradiated at recurrence at Gustave Roussy were included in this study. Parents and guardians gave their inform consent for the data collection and their analysis in this retrospective study that was approved by the Institutional Review Board (Commission Scientifique des Essais Thérapeutiques—CSET). Patients with multiple re-irradiations were also analyzed. Re-irradiation was discussed on a case-by-case basis by the internal multidisciplinary tumor board. Re-irradiation was decided on the ground of the re-irradiation strategies described in adults for glioblastoma (Nieder et al. 2008). Restriction criteria for re-irradiation were: poor clinical status of the patient, critical structures in the vicinity of the target, the estimated risk of radionecrosis taking into account any surgical injury and the precocity of the relapse.

### Definitions

Gross-total resection (GTR) was defined as no evidence of disease on postoperative neuroimaging and subtotal resection (STR) if there was evidence of residuum. The extent of surgery was judged by the analysis of the operative report with the post-operative imaging performed within 72 h. In case of discrepancy between neurosurgeons and radiological review, the priority was given to the imaging. For 6 patients where postoperative neuroimaging within 72 h was not available (n = 5) or not interpretable because of spinal surgery with osteosynthetic material implanted (n = 1), quality of surgery was established by the neurosurgeon’s report only.

Local relapse included solely relapse at the primary site. Metastatic relapses included relapse at sites not previously involved with tumor. Patients with combined relapse were analyzed in metastatic group.

For the analyses, the types of re-irradiation were grouped according to two opposite intention to treat: on one hand, the focal approach where patients received radiotherapy limited to the involved field of relapse with a conformal technique, and on the other hand, the prophylactic approach where children were treated with larger fields of irradiation to cover the involved site as well as previously untreated sites within the limitations due to the first irradiation.

### Statistical analyses

Time to progression was measured from the initiation of first course of radiotherapy (TTP1) or from the initiation of second radiotherapy course (TTP2) to the neuroimaging-documented time of relapse or progression. Progression free survival (PFS) was defined as the time interval from the initiation of radiotherapy course to date of tumor relapse or progression or last follow up. Overall survival (OS) was defined as the time interval from the initiation of radiotherapy course to death from any cause or last known date of survival. OS and PFS were analyzed using Kaplan–Meier curves. Comparison of PFS and OS between the different treatments was examined with the log-rank test. Statistical analyses were performed using SPPS 19^®^ software.

## Results

### Patients

**Fig. 1:**
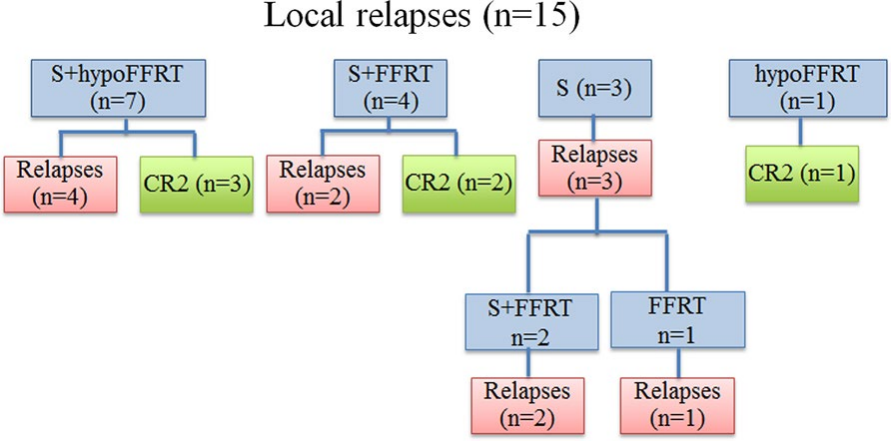
Influence of radiotherapy techniques on local relapses control. *S* Surgery, *CR* complete remission; Surgery in 8 patients treated with hypoFFRT: 7/8 (7 GTR); Surgery in 7 patients treated with FFRT: 6/7 (4 GTR, 2STR)

**Fig. 2:**
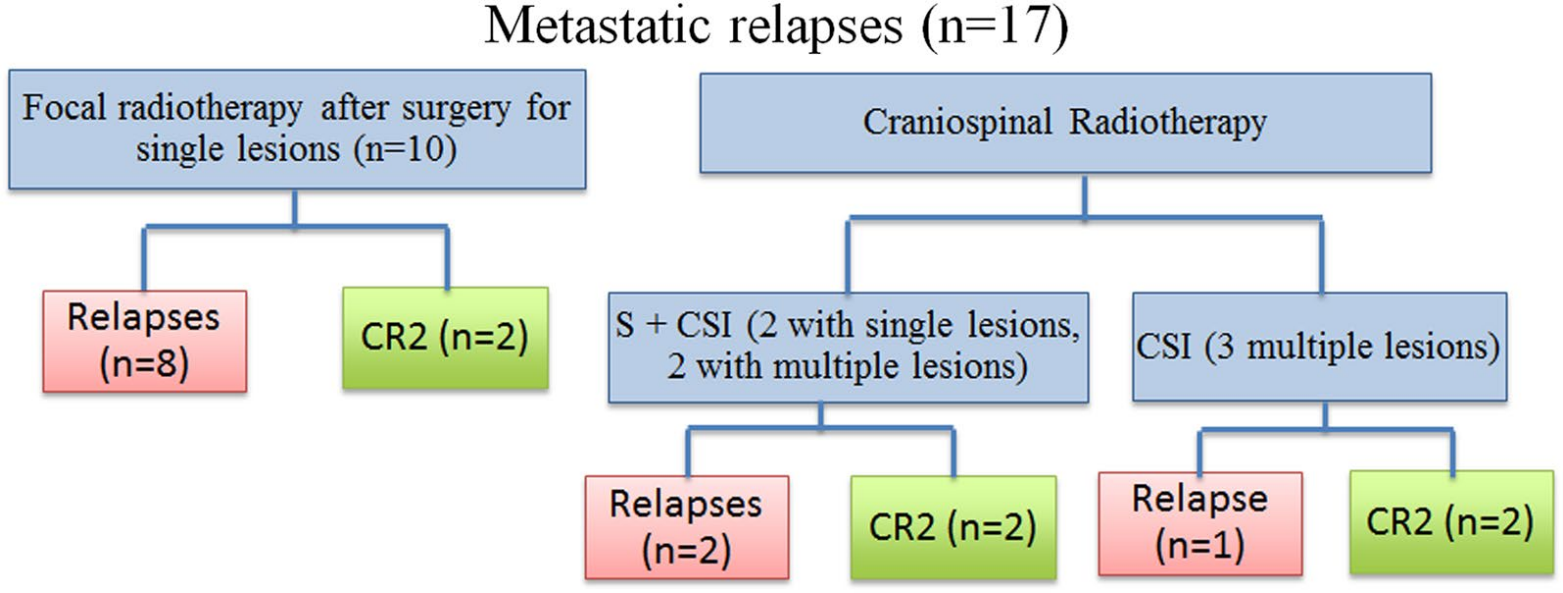
Influence of radiotherapy techniques on metastatic relapses control. *S* Surgery, *CR* complete remission; Surgery in 10 patients with focal radiotherapy: 10/10 (5 GTR and 5 STR); Surgery in 7 patients with CSI: 4/7 (1 GTR and 3 STR)

**Fig. 3:**
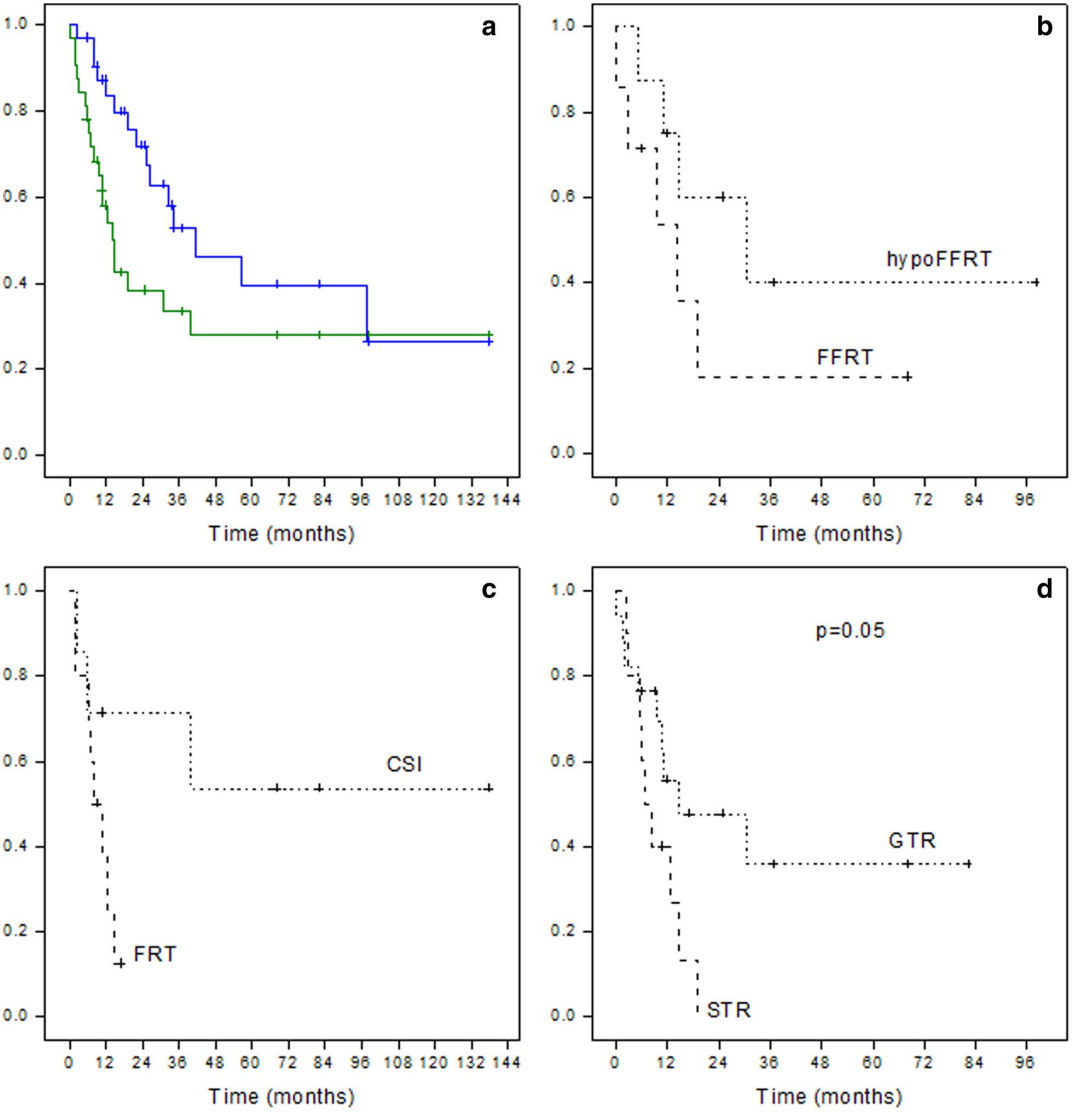
Kaplan–Meier survival curves showing Progression free survival and Overall Survival (**a**) of all patients and Progression free survival according to type of radiotherapy for local relapses (**b**) and metastatic relapses (**c**). Kaplan–Meier survival curves showing Progression Free Survival after radiotherapy 2 according to quality of surgery before radiotherapy 2 (**d**). *CSI* craniospinal irradiation, *FFRT* focal fractionated radiotherapy, *FRT* focal radiotherapy (HypoFFRT, FFRT and spinal radiotherapy), *HypoFFRT* hypofractionated radiotherapy. *GTR* gross total resection, *STR* subtotal resection. Surgery 1 b: hypo FFRT (7 GTR, 1 NS), FFRT (4 GTR, 2 STR, 1 NS). Surgery 1 c: CSI (1 GTR, 3 STR, 3 NS), FRT (5 GTR, 5 STR)

32 pediatric patients were re-irradiated at the time of recurrence. During the same period, twenty-two patients were not re-irradiated at relapse. In the more recent years, the proportion of patients re-irradiated at relapse increased as long as feasibility and safety were ascertained (data not shown). Clinical information and outcomes are listed in Tables 1 and 2. Figures 1 and 2 describe the treatment received for local and metastatic relapses, respectively. Median age was 6.2 years (0.4–17.5) at diagnosis and 8.2 years (3.1–18.6) at second irradiation (RT2). Female/Male ratio was 0.88 (15/17). Median time to progression from RT1 was 1.2 years (0.2–8.3) and median time to progression from RT2 was 0.7 years (latest progression registered at 3.3 years). Median time from initiation RT1 to initiation RT2 (Interval RT1-RT2) was 2.1 years (0.5–8.8). Median PFS and OS for all patients after RT2 were 1.2 and 3.5 years ( Fig. 3a). PFS and OS after RT2 according to the type of relapse (local vs metastatic) were 1.2 versus 1.1 years and 4.7 versus 3.4 years respectively (p = NS).

**Table 1:** Patient´s characteristics at baseline and before re-irradiation

Patient	Age at diagnosis (years)	Tumor location	Histology (grade)	Treatment at diagnosis	Chemotherapy before RT1	Surgery before RT1	Radiotherapy 1 (dose Gy)	Pattern of failure	Lesion	TTP 1 (months)
1	6.6	IT	AE (III)	No RT	Yes	GTR	FFRT (54)	Local	S	50
2	1.1	IT	AE (III)	No RT	Yes	GTR	FFRT (57.6)	Local	S	34
3	1.6	IT	AE (III)	RT	No	GTR	FFRT (56)	Local	S	31
4	7.7	IT	AE (III)	RT	No	GTR	FFRT (54)	Local	M	43
5	7.6	ST	AE (III)	RT	No	GTR	FFRT (54)	Local	S	12
6	2.0	ST	AE (III)	No RT	Yes	GTR	FFRT (54)	Local	S	18
7	3.7	IT	AE (III)	No RT	Yes	GTR	FFRT (54)	Local	S	26
8	11.2	IT	AE (III)	RT	Yes	STR	FFRT (59.4)	Local	M	33
9	11.4	ST	AE (III)	RT	No	GTR	FFRT (54)	Local	S	11
10	10.3	IT	AE (III)	RT	No	GTR	FFRT (54)	Local	S	61
11	2.4	ST	AE (III)	No RT	Yes	GTR	FFRT (54)	Local	S	13
12	5.2	ST	AE (III)	No RT	No	GTR	FFRT (54)	Local	M	10
13	7.6	IT	AE (III)	RT	No	GTR	FFRT (54)	Local	S	18
14	2.7	IT	E (II)	RT	Yes	STR	FFRT (50.4)	Local	S	86
15	2.2	IT	E (II)	No RT	Yes	NS	SRS (18)	Local	S	36
16	0.4	IT	AE (III)	No RT	Yes	GTR	FFRT (48.6)	Metastatic	S	65
17	2.6	IT	AE (III)	RT	No	GTR	FFRT (59.4)	Metastatic	S	12
18	0.8	IT	AE (III)	No RT	Yes	GTR	FFRT (54)	Metastatic	S	3
19	9.8	IT	AE (III)	RT	No	GTR	FFRT (54)	Metastatic	S	45
20	6.1	IT	AE (III)	RT	No	STR	FFRT (55)	Metastatic	S	7
21	14.6	IT	AE (III)	RT	No	GTR	FFRT (54)	Metastatic	S	10
22	5.5	IT	AE (III)	RT	No	STR	FFRT (50)	Metastatic	S	4
23	6.3	IT	AE (III)	RT	No	GTR	FFRT (59.4)	Metastatic	S	14
24	4.0	IT	AE (III)	No RT	Yes	STR	FFRT (55)	Metastatic	S	4
25	6.8	IT	E (II)	RT	No	GTR	FFRT (54)	Metastatic	S	8
26	17.1	SP	AE (III)	RT	No	STR	FFRT (54)	Metastatic	M	2
27	8.9	IT	E (II)	RT	No	STR	FFRT (55)	Metastatic	S	100
28	3.7	IT	E (II)	RT	No	GTR	FFRT (50.4)	Metastatic C	M	13
29	7.3	SP	E (II)	RT	No	STR	FFRT (41.4)	Metastatic C	M	8
30	1.8	IT	AE (III)	RT	No	GTR	FFRT (54)	Metastatic	M	11
31	16.1	SP	E (II)	RT	Yes	STR	FFRT (55)	Metastatic	M	25
32	6.6	IT	AE (III)	RT	Yes	STR	FFRT (54)	Metastatic	S	15

*AE* anaplastic ependymoma, *E* ependymoma, *FFRT* focal fractionated radiotherapy, *GTR* gross total resection, *IT* infratentorial, *Metastatic C* metastatic combined, *M* multiple, *NS* no surgery, *RT* radiotherapy, *S* single, *SP* spinal, *ST* supratentorial, *STR* subtotal resection, *SRS* stereotactic radiosurgery, *TTP1* time to progression after first radiotherapy

**Table 2:** Patient´s characteristics at the time of re-irradiation and outcomes

	RT1-RT2 (months)	RT1-failure-RT2 (months)	Chemotherapy before RT2	Surgery before RT2	RT2 total Gy; dose per fraction	Site RT2	RT2 failure	Location RT2 failure*	TTP 2 or last follow up (months)	FU after RT2 (months)	Current status
Local relapses											
*HypoFFRT*											
1	52	2	No	GTR	35; 3.5	4th ventricle	No	DA	25	25	NED
2	37	3	No	GTR	44; 4.4	4th ventricle	Yes	Metastatic	11	34	NED
3	35	3	No	GTR	35; 3.5	4th ventricle	Yes	Infield	31	31	PD
4	46	2	No	GTR	27; 4.5	4th ventricle	Yes	Metastatic	15	57	DOD
5	16	4	No	GTR	35; 3.5	Left parietal	Yes	Margin/mets	5	12	DOD
6	22	3	No	GTR	25; 5	Left parietal	No	DA	37	37	NED
7	27	1	No	NS	14	Left cerebellar	No	DA	99	99	NED
8	34	1	No	GTR	36; 6	4th + Left lateral V	No	DA	12	12	NED
*FFRT*											
9	44	33	Yes	STR	50.4; 1.8	Left temporal	Yes	Infield	19	27	DOD
10	70	9	No	NS	54; 1.8	Right CP angle	Yes	Infield	14	24	NED
11	31	18	No	GTR	DA	Parietal	Yes	Infield	0	2	DOD
12	12	3	Yes	STR	37.5; 2.5	Fronto-parietal	Yes	Margin/mets	3	19	DOD
13	23	5	No	GTR	45; 1.8	4th ventricle	Yes	Infield/margin	9	35	DOD
14	88	2	No	GTR	59.4; 1.8	Temporo-parietal	No	DA	6	6	NED
15	38	2	No	GTR	54; 1.8	4th ventricle	No	DA	69	69	NED
Metastatic relapses											
*HypoFFRT*											
16	69	4	No	GTR	51; 5.1	Right temporal	Yes	Metastatic	2	15	DOD
17	17	5	No	GTR	45; 3	Frontal	No	DA	17	17	NED
18	19	17	No	GTR	37.5; 6.25	Left lateral ventricle	No	DA	9	9	NED
*FFRT and spinal RT*											
19	51	6	Yes	STR	54; 2.25	Suprasellar	Yes	Metastatic	7	33	DOD
20	9	2	No	STR	35 + 15; 1.8	SP(B L3-TS)	Yes	Metastatic	8	22	DOD
21	12	2	Yes	GTR	54; 1.8	Fronto-parietal	Yes	Metastatic	2	35	DOD
22	8	3	No	STR	35 + 18; 1.8	SP(B TS)	Yes	Metastatic	15	42	DOD
23	15	1	No	GTR	54; 1.8	L4-S3	Yes	Metastatic	11	18	NED
24	6	2	Yes	STR	35 + 10; 1.8	SP(B T9-L1)	Yes	Metastatic	6	9	DOD
25	10	2	No	STR	36 + 14; 1.8	SP(B TS)	Yes	Metastatic	13	25	DOD
*CSI*											
26	15	13	Yes	NS	40 + 15; 1.8	BR (B PF)	No	DA	140	140	NED
27	105	5	No	GTR	36 + 14; 1.8	BR(PPF)/SP(B TS)	No	DA	83	83	NED
28	14	1	Yes	STR	36; 1.8	BR (PPF)/SP	Yes	Metastatic	2	8	DOD
29	35	27	No	NS	45/36; 1.8	BR/SP	Yes	Infield	40	99	DOD
30	16	4	Yes	STR	30/36 + 18; 1.8	BR(PPF)/SP(B C)	No	DA	11	11	NED
31	27	2	No	NS	40; 1.8	BR/SP to L1	No	DA	69	69	NED
32	17	2	Yes	STR	36 + 9; 1.8	BR (PPF)/SP(B C)	Yes	Metastatic	6	8	DOD

*B* boost, *BR* brain, *CSI* craniospinal irradiation, *C* conus, *DA* does not apply, *DOD* dead of disease*, FFRT* focal fractionated radiotherapy, *FU* follow up, *GTR* gross total resection, *HypoFFRT* hypofractionated stereotactic radiotherapy, *Mets* metastatic, *NED* no evidence of disease, *NS* no surgery, *PD* progressive disease, *PF* posterior fossa, *PPF* protection posterior fossa, *STR* subtotal resection, *SP* spinal axis, *TTP* time to progression, *TS* thecal sac, *V* ventricule

* Location RT2 failure regarding RT2 field infield at margin and metastatic

### Treatment before first course of radiotherapy (RT1)

After initial surgery, strategies containing radiotherapy (RT) or not containing radiotherapy (NRT) were offered according to the treatment period and age.

10 patients were treated in the NRT group: 9 patients underwent adjuvant chemotherapy, mainly BBSFOP (Grill et al. 2001) and UKCCSG infant protocol (Grundy et al. 2007) and 1 patient did not receive any adjuvant treatment. In these cases, first radiotherapy course (RT1) was performed at first progression or recurrence. Therefore, re-irradiation in this group corresponded to the second progression or recurrence. Efficacy and toxicity of re-irradiation was not different in these ten patients compared to the rest of the patients.

22 patients had radiotherapy after initial surgery. Three had chemotherapy before radiotherapy: one a cisplatin based-regimen, one high-dose chemotherapy due to misdiagnosis of PNET and one irinotecan-bevacizumab in an attempt to facilitate surgery in an aim to normalize tumor vascularization and minimize bleeding.

For all patients but one, RT1 was performed using conventional fractionation (1.8–2 Gy/session) with a median dose of 54 Gy (41.4–59.4). One was treated with SRS (Gammaknife™) 18 Gy (Table 1).

### Treatment at relapse after first course of radiotherapy (RT1 relapse)

Location of relapse was local in 15 patients and metastatic in 17 (two of them had also local relapses) (Table 1; Figs. 1, 2).

### PFS after second course of radiotherapy is greater than PFS after first course of radiotherapy in 50 % of patients

Fifteen patients achieved greater PFS after RT2 (PFS2) than after RT1 (PFS1). We compared the group of patients with PFS2 ≥ PFS1 and PFS2 < PFS1. We did not find statistically significant differences in the two groups with respect to age, location, type of relapse, type of radiotherapy, quality of surgery, strategy of treatment at diagnosis, delay to use RT2 after RT1 failure and interval RT1-RT2.

### Influence of radiotherapy techniques on local relapses control (Fig. 1)

Eight patients were treated with hypoFFRT. Five patients remain in complete remission (CR) with a median follow up of 2.6 years (1.0–8.3). Four patients relapsed, only one inside the field of re-irradiation (Table 2).

Seven patients were treated with FFRT. Three had experienced a first local relapse after RT1 treated only by surgery. Three patients remain in CR (one of them after RT2 relapse treated by single surgery). Five patients had a recurrence, four in the field and one at distant site. Patient 11 died at the beginning of RT2 due to rapid tumor progression.

Median PFS after RT2 for patients treated with hypoFFRT was 30.6 versus 14.3 months for patients treated with FFRT (p = 0.20) (Fig. 3b).

### Influence of radiotherapy techniques on metastatic relapses control (Fig. 2)

Treatment received is described in Fig. 2. Median PFS after RT2 was 0.7 years (0.2–1.1) for patients treated with local radiotherapy (hypoFFRT, FFRT, spinal radiotherapy) versus 6.8 (2.9–10.8) for patients treated with CSI (p = 0.073) (Fig. 3c).

### Benefit of surgery before re-irradiation

All children but 5 underwent a surgery. The 5 patients that did not underwent a resection had multiples sites and/or small lesions for which surgery was considered too risky; among these 5 children not re-operated, two were re-irradiated with FFRT and SRS for local relapses and 3 with CSI for metastatic relapses. Seventeen patients underwent GTR before RT2 and 10 STR. PFS after RT2 was better for the 17 patients who underwent GTR compared to the 10 patients where only a STR was possible with a median PFS2 of 14.7 versus 6.7 months (p = 0.05) (Fig. 3d). Of note however, 4 of 5 patients without resection before RT2 are alive (2 in CR and 2 with stable disease) with follow up ranging from 24 to 140 months. Three of these four survivors received craniospinal re-irradiation.

### Feasibility and results of third course of radiotherapy

Seven patients underwent a third course of radiotherapy (Patients 2, 4, 12, 13, 19, 22 and 23). All but one had relapsed at metastatic sites after RT2. They all had surgery before a third course of local radiotherapy (4 hypoFFRT and 3 FFRT). Four experienced distant and 1 local relapse after RT3. Only two are alive with a follow up of 6 and 20 months.

### Toxicity of re-irradiation

Five patients (4, 7, 10, 18 and 19) experienced radionecrosis as defined in the paper of Meyzer et al. (2010). Median interval RT1-RT2 for these patients was 45.6 versus 24.9 months for all others. Cumulated doses were not different from those of the patients that did not experience radionecrosis. Four presented asymptomatically and the symptoms of the remaining patient were resolutive within 2 weeks of steroids.

Among the fifteen survivors in CR, ten patients are attending school and three adult patients are working, one patient had troubles for schooling and we have no data for one patient. Lansky and Karnofsky performance status were above 80 % for all patients.

## Discussion

Re-irradiation has proven to be effective in this series owing the benefit in terms of PFS. Some groups suggest to take into account interval RT1-RT2 when offering re-irradiation (Merchant et al. 2008; Kano et al. 2010). Indeed, in our series 50 % of re-irradiated recurrent ependymoma achieved second PFS longer than first. Interval between RT1-RT2 did not predict PFS2 suggesting that even patients with early relapse after RT1 could benefit from re-irradiation. Of note, we did not re-irradiate children before 6 months after radiotherapy.

In contrast with the literature, for local relapses, patients treated by hypoFFRT achieved better, although not statistically significant, outcomes than those treated by FFRT. We observed local control in 6 (75 %) of patients treated with hypoFFRT versus 2 (28 %) by FFRT. Conversely, excellent disease control rate for local recurrences re-treated with FFRT have been reported by others (Merchant et al. 2008). Otherwise, Stauder et al. (2012) reported also good OS and local control rates for patients treated by SRS with smaller treatment volumes. Radionecrosis has been reported as a dreadful complication of SRS. Hoffman has reported 6 cases of radionecrosis (3 needed treatment) in a series of 12 patients treated by SRS (Hoffman et al. 2014). However, other group like in our report described low rate of radionecrosis with prompt resolution of symptoms with steroids (Merchant et al. 2009; Lo et al. 2006). There could be room for a randomized trial exploring long-term disease control and toxicities in children with isolated local relapses comparing FFRT with hypoFFRT.

Considering that CSI did not improve disease control in ependymoma at diagnosis (Vanuytsel and Brada 1991), some of our patients received focal radiotherapy for isolated metastasis. The finding of paramount importance in this study is the lack of long-term disease control of patients undergoing focal techniques (hypoFFRT, FFRT, spinal RT) for isolated metastatic relapses, especially for patients with spinal metastasis of an infratentorial tumor compared with a better control for patients treated for metastatic recurrences receiving CSI, even with more extensive disease at relapse. Most of the failures of re-irradiation were outside the radiation field, especially for those treated with a hypoFFRT, suggesting that CSI could improve disease control at this stage as well. To reinforce this idea, we also observed two patients with metastatic relapse re-irradiated by CSI excluding posterior fossa field who experienced posterior fossa RT2 relapse. Good disease control was achieved in patients with metastatic relapses re-treated by CSI even without recourse of surgery of the metastases proved elsewhere to be associated with a better prognosis. Merchant et al. reported a series of 12 patients with metastatic relapse treated by CSI with OS 100 % at 5 years and 9 of 12 patients without relapse after RT2 with a median follow up of 22 months (range 3–69) (Merchant et al. 2008). Bouffet et al. (2012) also reported good results in his series with all patients treated by CSI due to metastatic disease (n = 4) alive at a median follow up of 1.9 years (range 0.6–4.7). Our data showing the lack of efficacy of local irradiation of isolated metastasis speak in favor of a systematic craniospinal re-irradiation without PF sparing that was more efficient also in the two other cases series reported (Bouffet et al. 2012; Merchant et al. 2008).

Among the 27 patients re-operated before RT2, we found better outcome for patients who underwent GTR. The extent of surgery in the management of ependymoma at diagnosis is the strongest prognostic factor (Merchant et al. 2009; Pollack et al. 1995) and it has therefore been strongly recommended to re-operate the lesions at relapse whenever possible (Boop and Sgouros 2009). Merchant and Bouffet advocated the recourse to metastasectomy before re-irradiation but there is no data showing benefit of this strategy. All children that could be re-operated (single lesions or multiple lesions in the same field) were re-operated and for those where complete surgery could be achieved, the outcome was significantly better. Our data suggest therefore for the first time that even at relapse, the extent of surgery has a determinant role in prognosis.

We did not observe major toxicities linked to re-irradiation. Patients with radionecrosis did not have shorter intervals between RT1 and RT2. Impact of interval RT1–RT2 on the occurrence of radionecrosis is controversial. More recent studies do not observe correlation between interval RT1–RT2 and the incidence of radionecrosis (Hoffman et al. 2014).

In conclusion, re-irradiation at relapse is a feasible and potentially curative treatment since 15 patients are alive disease free after RT2 with a median follow-up of 2.1 years (range 0.5–11.6 years). Metastatic relapses may require CSI for better disease control. For local relapses, a randomized trial is warranted to compare hypoFFRT versus FFRT. For patients with isolated metastasis or local relapses, the complete resection of the lesion may offer a benefit in terms of disease control.

This study provides valuable preliminary information on the long-term evolution of children with re-irradiated recurrent ependymoma and findings should be confirmed at a multi-institutional level to propose changes in the treatment paradigms applied at relapse for ependymomas.
